# Under-diagnosis of alcohol-related problems and depression in a family practice in Japan

**DOI:** 10.1186/1447-056X-7-3

**Published:** 2008-09-29

**Authors:** Kenshi Yamada, Tetsuhiro Maeno, Kazuhiro Waza, Takeshi Sato

**Affiliations:** 1 Department of Psychiatry, Tokyo Metropolitan Geriatric Hospital, Tokyo, Japan; 2 Institute of Clinical Medicine, University of Tsukuba, Ibaraki, Japan; 3 Waza Clinic, Chiba, Japan; 4 Health Care Center, Saga University, Saga, Japan

## Abstract

**Aim:**

The aim of this survey was to assess the accuracy of a family physician's diagnosis of depression and alcoholism.

**Methods:**

Consecutive new adult patients attending a family practice in Japan between April 2004 and August 2006 were enrolled. Excluded were those with dementia or visual disturbance, and emergency cases. Participants completed a questionnaire regarding their complaints and socio-demographics. A research nurse conducted the Japanese version of the Mini-International Neuropsychiatric Interview (J-MINI) in the interview room. The doctor independently performed usual practice and recorded his own clinical diagnoses. A researcher listed the clinical diagnoses and complaints, including J-MINI or clinically-diagnosed alcoholism and depression, using the International Classifications for Primary Care, Second Edition (ICPC-2) and calculated kappa statistics between the J-MINI and clinical diagnoses.

**Results:**

Of the 120 adult first-visit patients attending the clinics, 112 patients consented to participate in the survey and were enrolled. Fifty-one subjects were male and 61 female, and the average age was 40.7 ± 13.2 years. Eight alcohol-related disorders and five major depressions were diagnosed using the J-MINI, whereas no cases of alcoholism and eight depressions were diagnosed by the physician. Clinically overlooked patients tended to have acute illnesses like a common cold. Concordance between the clinical and research diagnosis was achieved only for three episodes of Major depression, resulting in a kappa statistic of 0.43.

**Conclusion:**

Although almost half of the major depressions were identified, all alcoholism was missed. A mental health screening instrument might be beneficial in family practice, especially to detect alcoholism.

## Background

Alcohol-related problems and depression are highly prevalent in general practice [1]. More than half of all persons with alcohol-related problems obtain all of their care from their general practitioner. Hence, primary care has a considerable potential as a site for prevention of and interventions for alcoholism [2]. Screening and interventions for alcoholism in primary care has been demonstrated to reduce drinking [3].

However, depression and alcoholism often are under-diagnosed and untreated in family practice. For example, family physicians in Australia failed to identify 65% of CAGE-defined alcohol abuse [CAGE: an acronym of key words found in the following screening questions for alcoholism: "Have you felt that you need to **c**ut down on your drinking?" "Have people **a**nnoyed you by criticizing your drinking?" "Have you ever felt bad or **g**uilty about your drinking?" "Have you ever had a morning **e**ye-opener?" [4]]. Physicians in the United States (US) detected only 49% of the current alcohol problems diagnosed using the Diagnostic Interview Schedule (DIS) [5]. An international multi-centre study conducted by the World Health Organization (WHO) revealed that physicians recognize just 42% of all cases of Major Depression diagnosed by means of the Composite International Diagnostic Interview (CIDI) [6]. These studies examined the percentage of simple agreement between physicians' diagnosis and diagnostic tools, or assessed characteristics that influence the detection of mental illnesses. However, few studies have evaluated patient complaints and doctors' diagnoses as potential causes of diagnostic errors pertaining to mental health.

Consequently, the aims of this survey were: 1) to evaluate the accuracy of a family physician's diagnosis of depression and alcoholism using a validated screening instrument; and 2) to assess patient complaints and physician diagnoses that result in alcoholism and depression being overlooked in family practice.

## Methods

### Research settings

In Japan, almost everyone is covered by national health insurance and pay 30 percent of medical fee. Patients generally have the freedom to choose the health care provider that they feel best fits their needs without concerns regarding costs.

The survey was conducted of a single family practice. The physician had 22 years of clinical experience and had been well-trained in the diagnosis and treatment of depression. The clinic was a solo practice under private management, located in Matsudo City, a suburb of Tokyo, Japan. The number of patients seen daily in the clinic generally ranged between 80 and 110 (average number 95). Approximately 10 percent of the patients seen were there for their first visit, and 45 percent of the patients were adults. Research clinics were pre-arranged, so that a research nurse was available to perform structured interviews. Research clinics were set for every second and fourth Saturday from April to September 2004, every second Saturday from November 2004 to March 2005, and every second Tuesday and Saturday from September 2005 to August 2006. The survey was conducted over a total of 25 days within the study period.

### Subjects

All consecutive new adult patients attending the above-noted research clinics for the first time were enrolled in the study. To be eligible, a patient had to be 18 or more years old and had to provide informed consent. Patients were excluded if they had a high fever (≥ 38.0°C), or any condition requiring emergency management, such as impaired consciousness, extensive drug reactions or deep burns. Otherwise eligible subjects also were excluded if they were unable to complete the questionnaire for any reason (e.g., patients with language barriers, dementia or visual disturbance).

### Measurements

The survey was conducted in the following order. First, each new adult patient was asked to complete a written questionnaire in the waiting room. This questionnaire included questions on socio-demographics, in addition to the following question: "What is your problem? Please write the reason for your visit." Second, the Japanese version of the Mini-International Neuropsychiatric Interview (J-MINI) was conducted by a research nurse in the interview room. Third, the doctor performed his usual clinical practice and recorded his own clinical diagnosis on the face sheet of each patient's registration form. Note that the research interview conducted by the research nurse and the clinical evaluation performed by the physician were carried out independently. Finally, the reason for visit and the clinical diagnosis were classified according to the International Classifications for Primary Care, Second Edition (ICPC-2) by an investigator independent of the attending physician and research nurse. The degree of agreement between the results of the research interview and the clinical evaluation were estimated, in terms of the diagnoses of Major Depressive Episode and Alcoholism, using kappa statistics, calculated using SPSS, version 11.0J.

### Diagnostic tools

The MINI is an abbreviated, structured, diagnostic interview that requires an administration time of 10–20 minutes. It is designed to allow non-specialists who have received formal training to screen for certain psychiatric diagnoses. It conforms both to the *International Classification of Mental and Behavioral Disorders*, *Tenth Revision *(ICD-10) and the *Diagnostic and Statistical Manual of Mental Disorders, Fourth Edition *(DSM-IV), and has been validated relative to the *Structured Clinical Interview for DSM-III-R *(SCID) and the *Composite International Diagnostic Interview *(CIDI) [7]. The Japanese version of the MINI (J-MINI) also has been validated [8]. Prior to starting this survey, research nurses received practical training in the use of an instruction videotape, dealing with the use of the MINI, a training process which was comparable to the standard training packet used in the study validating the J-MINI.

The ICPC-2 is problem-oriented disease classification system developed by the World Organization of Family Doctors (WONCA). It has compatibility with the ICD-10, and health care providers can classify, using a single classification, three important elements of the health care encounter: the reasons for the encounter, the diagnoses or problems, and the process of care [9].

## Results

Of the 120 consecutive patients who fulfilled the inclusion criteria during the observation period, 8 patients were excluded (5 refused, and 3 due to high fever) and 112 patients (93.3% of all eligible) were enrolled. Among the 112, 51 (45.5%) were male and 61 were female, and the average age was 40.7 ± 13.2 years. Descriptive characteristics of the subjects are shown in Table 1.

**Table 1:** Descriptive characteristics of patients (n = 112)

Characteristics	No (%)
Age, mean years (SD)	40.7 (13.2)
Sex	
Male	51 (45.5)
Female	61 (54.5)
Marital status	
Single	22 (19.6)
Married or Common-law	79 (70.5)
Divorced	6 (5.4)
Widowed	4 (3.6)
Unknown	1 (0.9)
Occupational status	
Unemployed	29 (25.9)
Part-time job	13 (11.6)
Full-time job	65 (58.0)
In school	3 (2.7)
Unknown	2 (1.8)
Educational level	
Junior high school graduate	8 (7.1)
High school graduate	28 (25.0)
College graduate	36 (32.1)
University graduate or higher	39 (34.8)
Unknown	1 (0.9)

Eight alcohol-related disorders and five Major Depressive Episodes were diagnosed by research nurses using the J-MINI, while no alcoholism and eight depressive episodes were diagnosed by the doctor. Table 2 is a list of the complaints and clinical diagnoses among the patients with MINI-diagnosed alcohol-related disorders. Table 3 is a list of the MINI or clinically-diagnosed major depressive episodes, and of patient complaints. Almost all patients whose depression or alcoholism was clinically overlooked either had an acute illness, like a common cold, or had attended the clinic for the sole purpose of a check-up.

**Table 2:** Clinical diagnoses and patient complaints among MINI-diagnosed alcohol-related disorders

**Age and sex**	**MINI diagnoses**	**Clinical diagnoses (ICPC2)**	**Patient complaints (ICPC2)**
40 years, male	Current alcoholism	Upper respiratory infection (R74)	counseling with a preventive purpose(A98) and cough(R05)
39 years, male	Current alcoholism	Streptococcal pharyngitis(R72)	Cough(R05)
48 years, male	Current alcoholism	Upper respiratory infection (R74)	Common cold(R74) and cough(R05)
36 years, female	Current alcoholism and Obsessive-Compulsive disorder	Upper respiratory infection (R74) and migraine(N89)	Common cold(R74)
28 years, male	Current alcoholism and Hypomanic episode	Upper respiratory infection (R74)	Cough(R05) and runny nose(R07)
46 years, male	Current alcohol abuse	Hypertension(K86)	Fear of hypertension(K25)
29 years, male	Current alcohol abuse	Acute bronchitis(R78)	Cough(R05)
41 years, male	Current alcohol abuse	Upper respiratory infection (R74) and depression(P76)	Sputum(R25)

ICPC2; International Classifications for Primary Care, Second Edition

MINI: Mini-International Neuropsychiatric Interview

**Table 3:** MINI or clinically-diagnosed depression and patient complaints

Age and sex	MINI diagnoses	Clinical diagnoses (ICPC2)	Patient complaints (ICPC2)
62 years, female	Major depressive episode	Glossitis (D83) and upper respiratory infection (R74)	Common cold (R74)
27 years, female	Major depressive episode	Upper respiratory infection (R74)	Common cold (R74)
25 years, female*	**Major depressive episode and Agoraphobia**	**Depression (P76) and gastritis (D87)**	**Epigastria (D02)**
35 years, female*	**Major depressive episode**	**Depression (P76) dehydration (T11) and dizziness (N17)**	**Dizziness (N17), headache (N01) and loss of appetite (T03)**
31 years, male*	**Major depressive episode**	**Depression (P76), anxiety neurosis (P74) and fainting (A06)**	**Fainting (A06), thirst (T01) and loose bowel movements(D11)**
63 years, male	Agoraphobia	Depression (P76)	Fatigue (A04)
31 years, male	Hypomanic episode, Obsessive-Compulsive disorder and Agoraphobia	Depression (P76), dehydration (T11) and vomiting (D10)	Vomiting (D10), loss of appetite (T03) and feeling depressed (P03)
47 years, male		Depression (P76) and tension headache (N95)	Cough (R05), nausea (D09), headache (N01)
24 years, female		Depression (P76) and upper respiratory infection (R74)	Feeling ill (A05) and common cold (R74)
41 years, male	Current alcohol abuse	Depression (P76) and upper respiratory infection (R74)	Sputum(R25)

* Concordance between research and clinical diagnosis

ICPC2; International Classifications for Primary Care, Second Edition

MINI: Mini-International Neuropsychiatric Interview

Concordance between the MINI and physician diagnosis was limited to three patients with major depression. Table 4 is a cross table for major depression, comparing the MINI and family physician's diagnoses. The estimated kappa statistic for major depression was 0.43.

**Table 4:** Cross table of major depression between MINI and family physician (FP) diagnosis

		Major depressive episode (MINI)	
		
		Positive (%) (N = 5)	Negative (%) (N = 107)
Major depression (FP)	Positive (N = 8)	3 (60.0)	5 (4.7)
	Negative (N = 104)	2 (40.0)	102 (95.3)

FP; family physician, MINI; Mini-International Neuropsychiatric Interview

## Discussion

In this survey of a single family physician's practice, the kappa statistic, which measured the degree of agreement between the family physician and the J-MINI for major depression, was 0.43. Compared to the diagnostic accuracy by psychiatrists reported in J-MINI validation study by Otsubo and colleague [8], the family doctor in this survey had an acceptable level of diagnostic accuracy for major depression. Otsubo and colleagues reported that the kappa statistic estimating agreement between expert psychiatrists' diagnoses using diagnostic criteria and the J-MINI is 0.36 for major depression and 0.26 for alcoholism [8]. Spitzer and Fleiss used the kappa statistic to measure inter-observer agreement between psychiatrists before the era of research diagnostic criteria, and estimated a kappa of 0.41 for affective disorder (including neurotic or manic-depression) and 0.71 for alcoholism [10]. Even using the Structured Clinical Interview for DSM-III-R (SCID), kappa statistics estimated between mental health professionals were 0.42 for current major depression, and 0.76 for alcoholism, when they were used in general population [11]. For the Primary Care Evaluation of Mental Disorders (PRIME-MD), kappa statistics were 0.61 for major depression and 0.71 for alcoholism between primary care physicians and mental health professionals [12].

Because it corrects for chance agreement, the kappa statistic definitely is useful for calculating inter-observer concordance. A limitation, however, is that it depends upon disease prevalence or base rate. Consequently, estimates tend to be low when prevalence is low (especially below 10%), even if a high level agreement of agreement is observed [13-15]. Among the 112 patients screened in this survey, the rates of major depression and alcoholism were 4.5% and 7.1%, respectively. Both major depression and alcoholism have a reported prevalence of 3.7% among new adult patients in Japanese family practice, when the J-MINI is used [16]. Using the Diagnostic Interview Schedule (DIS), the prevalence among first-visit adult patients seen in general medicine outpatient clinics in Japan is reported to be 4.7% for major depression and 9.4% for alcoholism [17]. Among outpatients in hospital-based general practice in the US, the prevalence of major depression ranged from 7 to 19%, and of alcoholism from 3 to 7% [12]. The prevalence has been shown to vary dependent upon the diagnostic tools and the settings in which surveys are conducted.

The doctor in this survey, despite exhibiting an acceptable level of diagnostic accuracy for major depression, relative to experts, nonetheless missed all cases of alcoholism. Interestingly, almost every patient for whom a MINI-diagnosed mental health disorder was missed by the doctor had some acute illness, like a common cold or asthma. Because the reason for their visit was specific to physical symptoms, mental health problems might have been overlooked, except for the structured screening instrument. In typical medical practice, physicians often are not very successful diagnosing and treating alcohol-related problems or mental illnesses, likely due not only to their clinical skills, but also the patients' expectations and the constraints of the health care system [2]. In fact, even the MINI, which is the shortest structured psychiatric interview available, may be impractical in a busy clinical practice. Mental health screening may be required in primary care settings.

Our study does have several limitations. First, our results were obtained from a single clinical practice and the sample size was small. These two limitations restrict both the generalizability of our results and the confidence we can place upon them. Second, the physician who served as the attending in this study has extensive experience detecting depression in a primary care setting, and was a member of the research team, which may have led to a higher detection rate of depression than in most clinical practices. Third, convenience, rather than random, sampling was used for a variety of practical reasons, including the lack of research funding and our reluctance to over-burden staff. Further research is warranted using validated Japanese version self-report diagnostic tools, and a larger and more representative sample.

## Conclusion

In this survey, even though almost half of major depressive episodes were identified, a rate that is acceptable relative to previously-reported experts, all cases of alcoholism were missed. Because a detailed psychiatric interview might be impractical in a busy clinical practice, a mental health screening instrument might be beneficial in primary care settings, especially for alcohol-related problems

## Competing interests

The authors declare that they have no competing interests.

## Authors' contributions

KY participated in the study's design, conducted classifications using the ICPC-2 and all statistical analysis, and drafted the manuscript. KY and TM performed data collection and processing. KW participated in study design, especially with respect to ethical issues, and offered the research setting. TS and TM participated in study design and coordination and helped to draft the manuscript. All authors read and approved the final manuscript.

## Ethics approval

The Institutional Review Board of the Japanese Association of Family Medicine approved this research.
